# *The Syphilitic*—Dürer’s Woodcut, a Pandemic Unveiled

**DOI:** 10.3201/eid3107.AC3107

**Published:** 2025-07

**Authors:** Alexis Demas

**Affiliations:** Le Havre Hospital, Le Havre, France

**Keywords:** syphilis, bacteria, pandemic, The Syphilitic, Albrecht Dürer, Theodoric Ulsenius, art–science connection

**Figure:**
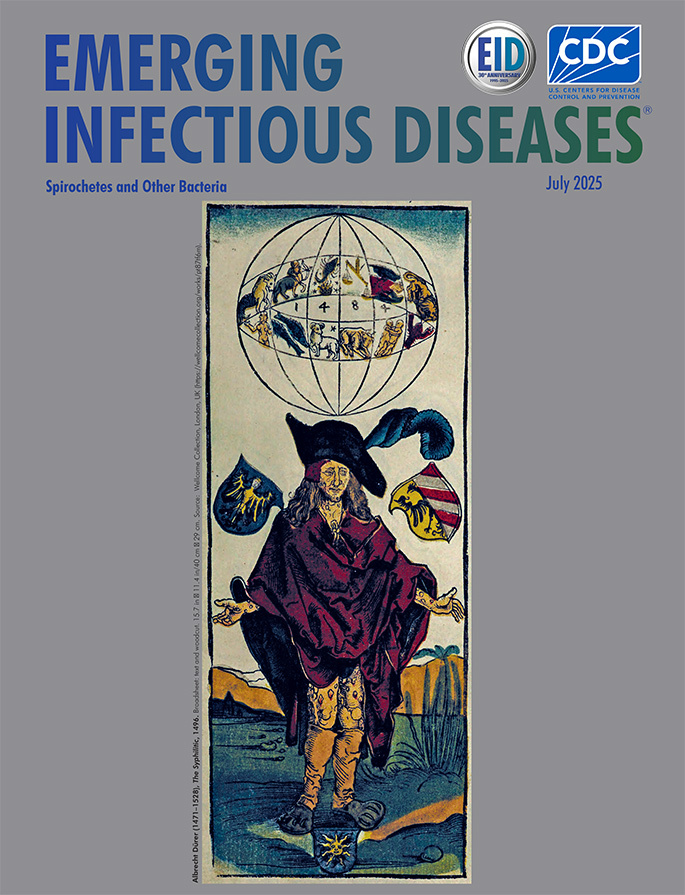
**Albrecht Dürer (1471–1528), *The Syphilitic*, 1496.** Broadsheet: text and woodcut. 15.7 in × 11.4 in/40 cm × 29 cm. Source: Wellcome Collection, London, UK (https://wellcomecollection.org/works/pt87tf6m).

In 1496, Albrecht Dürer, at the age of just 25, created a haunting woodcut to accompany a poem authored by German physician Theodoric Ulsenius. Entitled *The Syphilitic*, the image portrays a grotesque figure with bloated limbs, ulcerated skin, and a contorted face—an image both spiritual and pathological (Figure). Born in Nuremberg in 1471, Dürer would become one of the most influential figures of the Northern Renaissance, renowned for his technical mastery in engraving, his profound interest in humanist thought, and his innovative fusion of scientific observation and artistic expression. Although this woodcut dates to 1496, the prominent inscription of 1484 above the afflicted figure refers to a planetary conjunction that was, at the time, widely seen as an ominous sign. Dürer thus connects the astronomical event of 1484 with the outbreak of the disease he depicted 12 years later, framing it within a cosmological and prophetic context.

**Figure:**
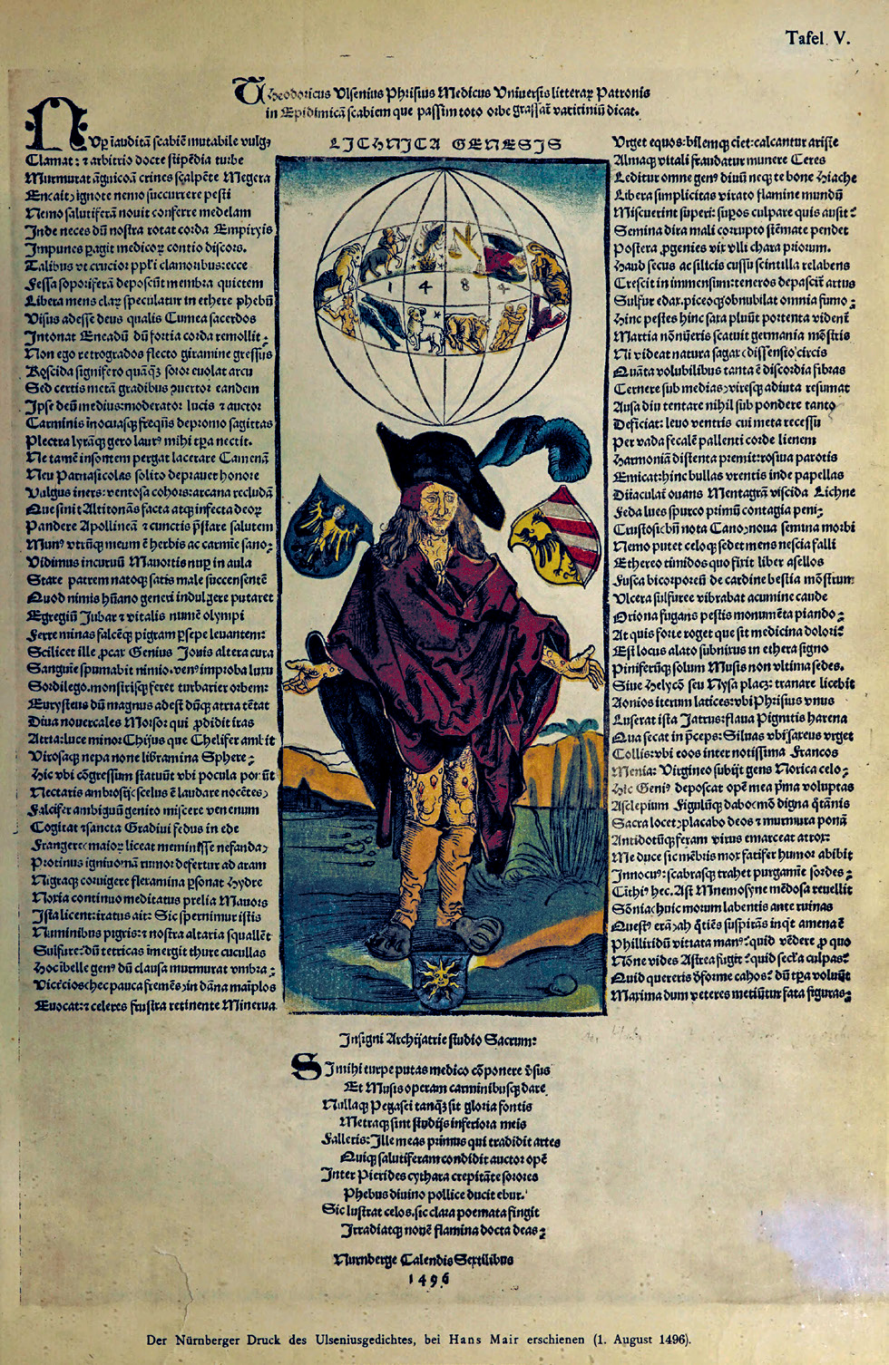
Full broadsheet of Albrecht Dürer’s *The Syphilitic*, showing text of poem authored by German physician Theodoric Ulsenius. Source: Wellcome Collection, London, UK (https://wellcomecollection.org/works/pt87tf6m).

This woodcut is one of the earliest known artistic representations of syphilis, capturing a moment when Europe was gripped by a new and devastating disease. Far beyond an artistic curiosity, this woodcut serves as a visual document—a Renaissance-era screenshot—preserving the medical, cultural, and existential shock of an emerging infectious disease. The accompanying Latin text was composed by Theodoricus Ulsenius, a German physician and humanist active in the late 15th Century. Although little is known about Ulsenius’s life, he is remembered for his medical writings and his interest in astrological explanations for disease, reflecting the intellectual currents of his era.

Syphilis, or the French disease as it was known in early texts, made its first explosive appearance in Europe in 1495, during the siege of Naples. The timing was no coincidence. Historical and microbiological evidence suggests that returning members of Christopher Columbus’s crew introduced a New World subspecies of *Treponema* (likely the agent of yaws), which rapidly evolved into the sexually transmitted *Treponema pallidum* in the environmental and social conditions of Europe. In a tragic irony of microbial globalization, this pathogen traveled eastward across the Atlantic while, simultaneously, European settlers from Europe carried diseases such as measles and smallpox westward, decimating the immunologically naive Native Americans. What emerged was an unintentional epidemic spread, waged not with intent, but with oblivious contact.

The image created by Dürer is imbued with dense and deliberate symbolism. The afflicted man stands prominently in the foreground, raising his hands toward the heavens in a gesture of despair and supplication, as if pleading for a divine explanation for his suffering. He is clothed in a loose, tattered robe, designed to conceal his skin and the visible lesions that emerge on the exposed areas: his face, legs, and hands. The garment, rendered in vivid red tones, accentuates his vulnerability and social isolation, the striking color drawing immediate attention. His emaciated features and hunched posture vividly evoke the physical devastation wrought by the disease. Behind him, an elaborate coat of arms displays a grotesque face, possibly symbolizing the French Disease. The accompanying Latin text by Ulsenius attributes the cause of the epidemic to a celestial phenomenon: the conjunction of Jupiter and Saturn in the sign of Scorpio. In an age when science, astrology, and theology were inseparably intertwined, such a cosmological explanation sought to make sense of a disease perceived as either divine punishment or an apocalyptic omen. Thus, Dürer’s composition serves not merely as a depiction of physical suffering but as a complex meditation on fate, human frailty, and the blurred boundaries between medical and metaphysical knowledge.

From a modern diagnostic perspective, the image invites an exercise in iconodiagnosis (the retrospective medical diagnosis carried out on a work of art depicting a human person). The skin lesions and facial features evoke differential considerations including leprosy, psoriasis, or even advanced scabies. But the timing of the image, just 1 year after the Europe outbreak, strongly favors a florid case of secondary or early tertiary syphilis. Dürer’s woodcut captures the grotesque morphology and rapid dissemination of a novel pathogen whose biologic behavior defied both Galenic medicine and moral explanation (the interpretation of disease as both a natural imbalance of the bodily humors, according to Galenic medical theory, and as a consequence of moral or sinful behavior).

Artists, often more sensitive to the undercurrents of their time than chroniclers or physicians, became inadvertent epidemiologists. Their works encode patterns of disease and perception. Just as Pieter Bruegel’s *The Triumph of Death* reflected the cultural impact of plague, Dürer’s syphilitic figure chronicles a microbial invasion frozen in time. Rather than depicting a microbial event in real time, Dürer’s woodcut offers a visual chronicle of a society grappling with a new and terrifying disease, blending observable symptoms with astrological and moral interpretations of human suffering. These images allow today’s physicians, historians, and scientists to revisit the past with clinical insight and to better understand how societies respond to emerging diseases.

Despite centuries of medical progress, syphilis remains with us. Recent epidemiologic reports from the World Health Organization show a rising global incidence of syphilis and other sexually transmitted infections, particularly among young adults and marginalized populations. This resurgence, despite the availability of effective antibiotics and preventive tools, prompts a reevaluation of our current strategies for public health education, access to care, and behavioral interventions.

The pandemic of the 1490s may seem distant, but it holds direct lessons for today. In both instances, newly mobile populations, evolving pathogens, and delayed institutional responses allowed infections to flourish. The human body, caught between biology and belief, becomes the ultimate witness to history.

Dürer’s syphilitic figure, though carved in wood more than 5 centuries ago, reminds us that microbes are protagonists in the story of civilization. Microbiological incursions are often sudden, the consequences lasting, and their documentation—through science and art—are essential to our understanding. In today’s era of emerging pathogens and renewed global vulnerability, this Renaissance woodcut stands not only as an artistic relic but as a timeless warning.
